# Cerebral blood flow from arterial spin labeling as an imaging biomarker of outcome after endovascular therapy for ischemic stroke

**DOI:** 10.1177/0271678X241267066

**Published:** 2024-10-04

**Authors:** Moritz R Hernandez Petzsche, Johannes Bürkle, Gabriel Hoffmann, Claus Zimmer, Sebastian Rühling, Julian Schwarting, Silke Wunderlich, Christian Maegerlein, Tobias Boeckh-Behrens, Stefan Kaczmarz, Maria Berndt-Mück, Nico Sollmann

**Affiliations:** 1 Department of Diagnostic and Interventional Neuroradiology, School of Medicine, Klinikum rechts der Isar, Technical University of Munich, Munich, Germany; 2 TUM-Neuroimaging Center, Klinikum rechts der Isar, Technical University of Munich, Munich, Germany; 3 Department of Neurology, School of Medicine, Klinikum rechts der Isar, Technical University of Munich, Munich, Germany; 4 Philips GmbH Market DACH, Hamburg, Germany; 5 Department of Diagnostic and Interventional Radiology, University Hospital Ulm, Ulm, Germany

**Keywords:** ASL, stroke, outcome, CBF, no-reflow phenomenon

## Abstract

Arterial spin labeling (ASL) is a contrast agent-free magnetic resonance imaging (MRI) technique to measure cerebral blood flow (CBF). We sought to investigate effects of CBF within the infarct on outcome and risk of hemorrhagic transformation (HT). In 111 patients (median age: 74 years, 50 men) who had undergone mechanical thrombectomy (MT) for ischemic stroke of the anterior circulation (median interval: 4 days between MT and MRI), post-stroke %CBF difference from pseudo-continuous ASL was calculated within the diffusion-weighted imaging (DWI)-positive infarct territory following lesion segmentation in relationship to the unaffected contralateral side. Functional independence was defined as a modified Rankin Scale (mRS) of 0–2 at 90 days post-stroke. %CBF difference, pre-stroke mRS, and infarct volume were independently associated with functional independence in a multivariate regression model. %CBF difference was comparable between patients with and without HT. A subcohort of 10 patients with decreased infarct-CBF despite expanded Treatment in Cerebral Infarction (eTICI) 2c or 3 recanalization was identified (likely related to the no-reflow phenomenon). Outcome was significantly worse in this group compared to the remaining cohort. In conclusion, ASL-derived %CBF difference from the DWI-positive infarct territory independently predicted functional independence, but %CBF difference was not significantly associated with an increased risk of HT.

## Introduction

Mechanical thrombectomy (MT) for large vessel occlusion (LVO) of the anterior circulation has revolutionized stroke care in the last decade.^1–5^ However, unfavorable clinical outcome is still frequent and image-based biomarkers to predict outcome are needed for clinical decision-making.^6^

Changes in brain hemodynamics caused by LVO can be measured using perfusion imaging.^7–9^ Specifically, LVO causes a reduction of cerebral blood flow (CBF) in the brain parenchyma located distally in relation to the occlusion, entailing the risk of tissue infarct.^10^ After MT, CBF in the infarct territory can vary widely from increased to persistently decreased, partly depending on the success of recanalization.^11–13^ As a gadolinium-free perfusion imaging method, magnetic resonance imaging (MRI)-based arterial spin labeling (ASL) uses blood-water of the brain-feeding arteries as an endogenous tracer to measure brain perfusion.^14–16^ Pseudo-continuous ASL (pCASL), a hybrid of the previously used and more artifact-susceptible techniques continuous ASL (CASL) and pulsed ASL (PASL), has been shown to provide sufficient signal-to-noise ratio (SNR) and wider clinical availability.^14,17,18^

In ischemic stroke, ASL has been shown to be highly sensitive to detecting perfusion changes following ischemic stroke for both anterior and posterior circulation stroke.^19,20^ Associations between brain perfusion increase derived from ASL and increased risk of post-stroke hemorrhagic transformation (HT) have also been demonstrated.^21,22^ Similarly, perfusion increase as measured by transcranial duplex sonography as well as MRI-based dynamic susceptibility contrast (DSC)-MRI has been associated with an increased risk of bleeding, infarct-related edema, and worse outcome after MT.^23^ Contrarily, associations between infarct perfusion increase measured by pCASL and lower National Institutes of Health Stroke Scale (NIHSS) scores 24 hours after stroke onset have been reported previously.^12^ A correlation between immediate post-stroke perfusion increase measured by PASL and higher rates of positive functional outcome measured by modified Rankin Scale (mRS) scores 90 days after stroke has also been shown.^13^ The no-reflow phenomenon, represented by diminished cerebral perfusion in a setting of insult despite successful revascularization therapy, has been postulated as part of the cause of the relationship between infarct CBF and clinical outcome.^24,25^

In this study, we aimed to evaluate pCASL-based CBF of the infarct core obtained few days after MT as a prognostic image-based biomarker. We also investigated the relevance of infarct perfusion increase by CBF for the development of HT in patients who underwent MT after anterior circulation stroke following LVO. We hypothesized that pCASL-based perfusion would allow prediction of clinical outcome. Furthermore, we sought to evaluate pCASL-based CBF measurements as a screening tool for the no-reflow phenomenon.

## Material and methods

### Study population

This mono-centric study, conducted in accordance with the Helsinki Declaration of 1975 and as revised in 1983, was approved by the institutional review board of the Faculty of Medicine of the Technical University of Munich (approval numbers: 28/19 S-SR/2019-28_1-S/2019-28_2-S) and analyzed prospectively acquired data. The basic study design included two time intervals for data acquisition per patient, namely the acute post-stroke phase (few days after stroke) and a follow-up (FU) phase (3 to 12 months after stroke), for which patients provided written informed consent. The MRI sequences needed for the present investigation were acquired as part of our clinical standard in-house scanning examinations performed few days after MT, with those post-stroke image acquisitions being primarily conducted for clinical indications.

**Figure 1.**
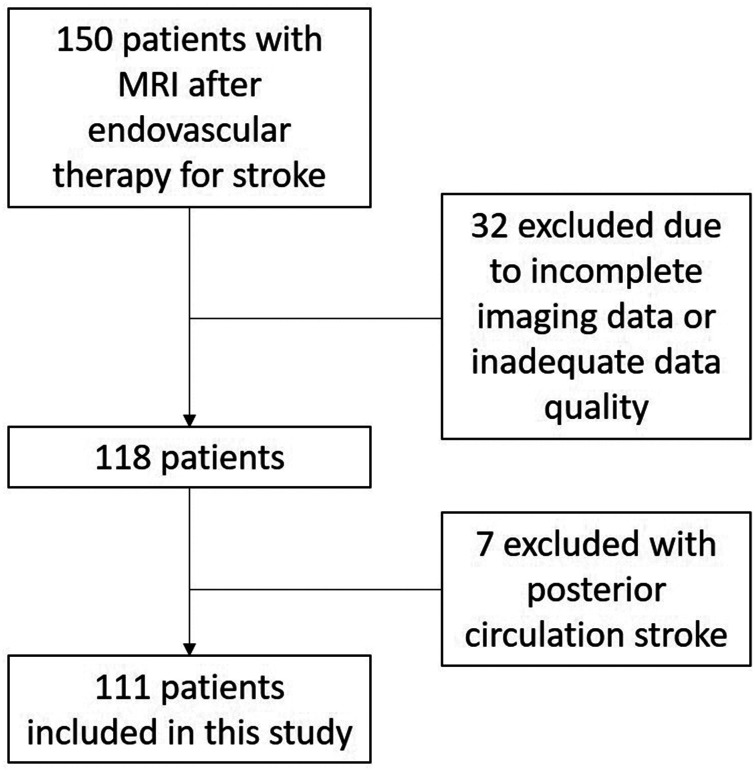
Flow chart of patient inclusion.

Between October 2020 and March 2022, a total of 150 consecutive patients received cerebral MRI after MT for ischemic stroke in our comprehensive stroke center. Inclusion criteria were age of at least 18 years and prior MT following ischemic stroke due to LVO (with a maximum interval of 10 days between MT and post-stroke MRI acquisitions). Exclusion criteria were pregnancy and contraindications for cerebral MRI (e.g., cochlear implants). Patients with inadequate image quality or incomplete imaging data were excluded from this study (n = 32). All patients with posterior circulation stroke were also excluded from this study (n = 7). Thus, the final study cohort was constituted of 111 patients (Figure 1). All MRI acquisitions were performed during the inpatient phase of stroke care.

### Clinical data and outcome measures

Clinical and imaging data as well as outcome measures were analyzed for this study. Basic demographic, clinical, and interventional data of patients were gathered and are shown in Table 1. Neurologists assessed the NIHSS scores at the time of admission.^26^ The mRS score was used to measure disability before stroke and at FU examinations 90 days post-stroke.^27^ Functional independence was defined as mRS ≤2 at 90-day FU examinations.^1–5^

**Table 1.** Cohort characteristics. All variables were compared between the subcohort with functional independence (mRS ≤ 2 at 3 months) and without functional independence (mRS > 2 at 3 months), central columns. Comparisons of all variables between the subcohort with no-reflow phenomenon and the rest of the cohort are shown in the three rightmost columns. All variable values were rounded to the nearest full digit. All p-values were rounded to the nearest 3rd decimal place.

Cohort characteristics	All (n = 111)	mRS ≤ 2	mRS > 2	p	No-reflow	Others	p
N=	111	65	46		10	101	
Age, median (IQR)	**74 (61–82)**	70 (58–80)	77 (66–84)	**0.036**	85 (71–91)	72 (61–81)	**0.016**
Male, n (%)	**50 (45)**	30 (46)	20 (44)	0.848	5 (50)	45 (45)	0.752
Pre-stroke mRS, median (IQR)	**0 (0–1)**	0 (0–0)	1 (0–3)	**<0.001**	1 (0–3)	0 (0–1)	0.286
NIHSS at admission, median (IQR)	**12 (7–16)**	10 (6–15)	15 (9–17)	**0.028**	14 (6–16)	12 (7–17)	0.921
ASPECTS, median (IQR)	**9 (7–10)**	9 (7–10)	9 (6–10)	**0.019**	9 (7–10)	9 (7–10)	0.879
Stroke to MRI in days, median (IQR)	**4 (3–5)**	4 (3–5)	4 (3–6)	0.143	6 (3–9)	4 (3–5)	0.057
Stroke risk factors, n (%)							
Hypertension	**78 (70)**	41 (63)	37 (77)	0.088	9 (90)	69 (69)	0.276
Diabetes mellitus	**21 (19)**	9 (14)	12 (26)	0.137	1 (10)	20 (20)	0.683
History of smoking	**33 (30)**	22 (34)	11 (24)	0.811	3 (30)	30 (30)	1.000
Previous stroke or TIA	**23 (21)**	11 (17)	12 (26)	0.241	2 (20)	21 (21)	1.000
Hyperlipidemia	**25 (23)**	12 (19)	13 (28)	0.249	4 (40)	21 (21)	0.238
Atrial fibrillation	**59 (53)**	33 (51)	26 (57)	0.562	4 (40)	55 (55)	0.730
Previous medication, n (%)							
Anticoagulation	**36 (32)**	17 (26)	19 (41)	0.145	1 (10)	35 (35)	0.158
Statin therapy	**35 (35)**	17 (26)	18 (39)	0.137	5 (50)	30 (30)	0.269
Platelet aggregation inhibitors	**30 (27)**	17 (26)	13 (28)	0.827	6 (60)	24 (24)	**0.018**
Pathogenesis, n (%)							
Large-artery atherosclerosis	**10 (9)**	5 (8)	5 (11)	0.738	1 (10)	9 (9)	1.000
Cardio-embolism	**55 (50)**	29 (45)	26 (57)	0.251	4 (40)	51 (51)	0.742
Small-vessel occlusion	**NA**	NA	NA	NA	NA	NA	NA
Stroke of other determined etiology	**7 (6)**	4 (6)	3 (7)	1.000	1 (10)	6 (6)	0.494
Stroke of undetermined etiology	**37 (33)**	26 (40)	11 (24)	0.101	4 (40)	33 (33)	0.729
SO or LSW to GRO in min, median (IQR)	**290 (184–562)**	275 (160–477)	318 (197–731)	0.240	472 (263–1155)	275 (180–543)	0.087
eTICI 2c or better, n (%)	**86 (78)**	54 (83)	32 (70)	**0.031**	10 (100)	76 (76)	0.209
eTICI 2 b or better, n (%)	**103 (93)**	60 (92)	43 (94)	0.649	10 (100)	93 (93)	1.000
Intravenous thrombolysis, n (%)	**47 (42)**	31 (48)	16 (35)	0.175	4 (40)	43 (43)	1.000
Infarct volume in ml, median (IQR)	**51 (13–136)**	33 (9–81)	112 (23–249)	**<0.001**	81 (15–224)	51 (12–133)	0.537
%CBF difference, median (IQR)	**16 (−9–51)**	21 (0–55)	3 (−23–44)	**0.042**	−37 (−51–(−18))	20 (−4–53)	**<0.001**
Hemorrhagic transformation, n (%)	**49 (44)**	24 (38)	25 (54)	0.082	5 (50)	44 (44)	0.747
HI1	**28 (25)**	15 (23)	13 (28)	0.658	3 (30)	25 (25)	0.711
HI2	**10 (9)**	4 (6)	6 (13)	0.314	1 (10)	9 (9)	1.000
PH1	**4 (4)**	3 (5)	1 (2)	0.641	0 (0)	4 (4)	1.000
PH2	**7 (6)**	2 (3)	5 (11)	0.124	1 (10)	6 (6)	0.494
SH	**3 (3)**	0 (0)	3 (7)	0.068	1 (10)	2 (2)	0.249
Hematoma volume of HI1 or HI2 in ml, median (IQR)	**2 (1–8)**	1 (0–3)	7 (1–26)	0.054	14 (2–26)	2 (1–7)	0.233
Hematoma volume of PH1 or PH2 in ml, median (IQR)	**32 (6–53)**	7 (4–32)	51 (32–277)	**0.028**	59 (n = 1)	27 (6–44)	0.223
Death in-hospital, n (%)	**5 (5)**	0 (0)	5 (11)	**0.011**	2 (20)	3 (3)	0.063
mRS at 3 months, median (IQR)	**2 (1–4)**	NA	NA	NA	5 (3–6)	2 (1–4)	**0.002**
Functional independence (mRS ≤ 2 at 3 months), n (%)	**65 (59)**	NA	NA	NA	2 (20)	63 (63)	**0.015**

P-values below 0.05, indicating statistical significance, are shown in bold.

IQR: interquartile range; mRS: modified Rankin Scale; ASPECTS: Alberta Stroke Program Early CT Score; NIHSS: National Institutes of Health Stroke Scale; TIA: transient ischemic attack; SO: symptom onset; LSW: last seen well; GRO: groin time; eTICI: expanded Treatment in Cerebral Infarction; CBF: cerebral blood flow; HI: hemorrhagic infarction; PH: parenchymal hematoma; SH: symptomatic hemorrhage; NA: not applicable; MRI: magnetic resonance imaging.

Stroke pathogenesis was determined according to the international Trial of ORG 10172 in Acute Stroke Treatment (TOAST) classification.^28^ Presence of atrial fibrillation was documented as well as other cardiovascular risk factors such as history of smoking, hypertension, hyperlipidemia, and diabetes mellitus. Pre-existing treatment with platelet-inhibiting drugs, lipid-lowering therapies, or anticoagulation was documented from patient charts. Administration of pre-interventional intravenous tissue-type plasminogen activator (tPA) thrombolysis was assessed.

The Alberta Stroke Program Early Computed Tomography Score (ASPECTS) was recorded based on admission cranial CT (Philips Ingenuity CT, 120 kVp, iDose^4^ reconstruction algorithm).^29^ The time of symptom onset to groin puncture was recorded. If the time of symptom onset was unavailable, last seen well to groin time was recorded. Following MT, achieving near complete or complete recanalization, as previously characterized by Gozy et al., was defined as a score of 2c or 3 according to the Extended Treatment in Cerebral Infarction (eTICI) scheme.^30,31^

### Post-stroke MRI acquisitions

Cerebral MRI acquisitions were performed after MT in supine position on a 3T MRI system (Achieva dStream; Philips Healthcare, Best, The Netherlands) using a 32-channel head coil and a consistent imaging protocol in all included patients. All patients were scanned during the acute in-hospital setting prior to discharge.

Three-dimensional (3D) non-contrast-enhanced T1-weighted (T1w) images were acquired with a repetition time (TR) = 9 ms, echo time (TE) = 4 ms, field of view (FOV) = 240 × 252 × 200 mm^3^, 267 sagittal slices, acquired voxel size = 1 × 1 × 1 mm^3^, and a scan duration of 2:26 min. Susceptibility-weighted imaging (SWI) was performed with TR = 38 ms, TE = 6 ms, FOV =235 × 193.88 × 170.25 mm^3^, acquired voxel size =0.75 × 0.75 × 1.5 mm^3^, 227 transversal slices, and a scan duration of 3:01 min. Diffusion-weighted imaging (DWI) was acquired using a single-shot spin-echo echo-planar imaging (EPI) sequence, resulting in one non-diffusion-weighted image (b = 0 s/mm^2^) and 32 diffusion-weighted images (b = 1,000 s/mm^2^, 32 non-collinear gradient directions) with the following parameters: TR = 5000 ms, TE = 78 ms, flip angle = 90°, voxel size = 2 × 2 × 2 mm^3^, FOV = 224 × 224 × 144 mm^3^, reconstruction matrix = 112, interslice gap = 0 mm, 72 transversal slices, and a scan duration of 5:36 min. A high-resolution 3D fluid-attenuated inversion recovery (FLAIR) sequence was obtained with the following parameters: TR = 4800 ms, TE = 276 ms, inversion time (TI) =1650 ms, FOV = 250 ×250 × 199.94 mm³, acquisition of 281 sagittal slices, voxel size of 1 × 1 × 1 mm³, and a scan duration of 3:55 min.

A pCASL sequence with a two-dimensional (2D) EPI readout was set up in accordance with recent recommendations.^14^ Imaging used the following parameters: label duration = 1800 ms, post-label delay (PLD) =2000 ms, TR = 4478 ms, TE = 11 ms, flip angle = 90°, SENSE factor = 2, EPI factor = 25, voxel size = 3 ×3 × 5.5 mm^3^, FOV = 210 × 210 × 120 mm^3^, reconstruction matrix = 80, interslice gap = 0.55 mm, 20 transversal slices, and a scan duration of 5:31 min. Additional proton density-weighted (PDw) M_0_ data were acquired for CBF quantification. The labeling plane was placed manually on a straight segment of the brain-feeding arteries, above the bifurcation of the carotid arteries. The image volume was placed so that it covered the whole brain of each patient.

### Image processing and post-stroke MRI analysis

Image processing of T1w and pCASL data used custom-built MATLAB scripts (Matlab 2021 b; The MathWorks Inc., Natick, MA, USA) and Statistical Parametric Mapping (SPM12; Wellcome Centre, London, UK). Image time series were motion-corrected and CBF (in ml/100 g/min) was quantified according to previous recommendations.^14^

Whole-brain CBF maps were automatically co-registered to native T1w space. Additionally, DWI and SWI data were co-registered to T1w space. Tissue probability maps were segmented using SPM, and gray matter (GM) masks were applied to CBF maps at a threshold of 0.6 according to previous work.^32^ Infarct segmentation of DWI-positive ischemic lesions was performed semi-automatically using ITK-Snap (version 3.8.0; http://www.itksnap.org/pmwiki/pmwiki.php).^33^ The two raters performing segmentation (MRHP, 3 years of experience; JB, 1 year of experience) were blinded to all other information including clinical outcome and CBF values. The FLAIR images were available to the raters for all cases to aid in exact infarct border masking and to avoid false-positive segmentation (e.g., due to signals in DWI sequences that were not related to ischemic areas). The CBF values were evaluated from the overlay of the infarct mask and the GM mask with a threshold at 0.6 (CBF_infarct mask_). The infarct mask was then mirrored across the midline and the CBF values were evaluated from the overlay of the mirrored mask and the GM mask (CBF_mirrored mask_), ensuring readout of pCASL data only within the GM. Voxel counts of the infarct masks were extracted and multiplied by the voxel size to arrive at the infarct volume (in ml). The relative CBF difference was calculated patient-wise from the infarct masks in relation to the mirrored masks in the unaffected hemispheres using the following formula:

$$\%\mathrm{CBF}\;\mathrm{difference}=\frac{{\mathrm{CBF}}_{\mathrm{infarct}\;\mathrm{mask}}-CBF_{\mathrm{mirrored}\;\mathrm{mask}}}{CBF_{\mathrm{mirrored}\;\mathrm{mask}}}\times 100\%$$

Computation of relative CBF has been performed previously to compensate for differences in absolute CBF values between individual patients.^12,34^

The SWI data were used to screen for HT by one reader (MRHP, 3 years of experience), who was blinded to clinical outcome as well as MT details or eTICI scores. If HT was found, it was classified into hemorrhagic infarction (HI) or parenchymal hematoma (PH) as HI1, HI2, PH1, and PH2 according to the European Cooperative Acute Stroke Study (ECASS) II classification criteria.^35^ Symptomatic hemorrhage (SH) was defined as a secondary neurological deterioration of ≥4 points on the NHISS scale and evidence of cerebral hemorrhage on imaging.^5^

**Figure 2.**
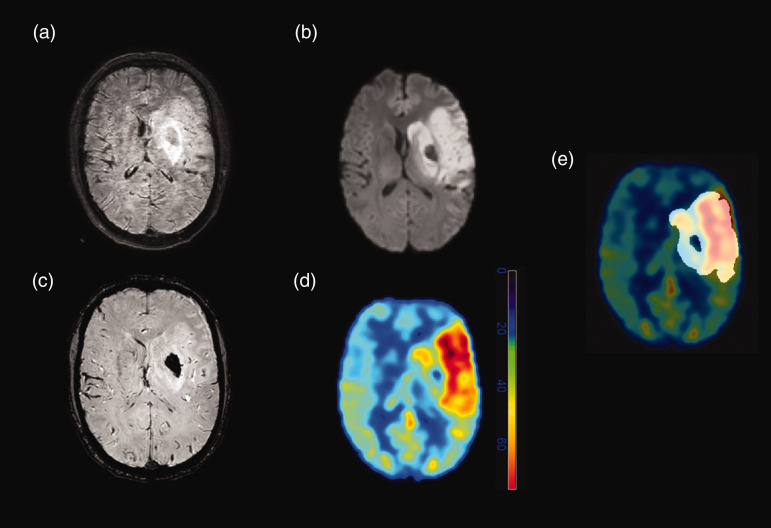
Magnetic resonance imaging (MRI) was acquired after left-sided thrombectomy of a thrombotic occlusion of the M1 segment of the middle cerebral artery in a 57-year-old male patient. (a) Fluid-attenuated inversion recovery (FLAIR) imaging (with moderate motion artifacts) shows left-sided edema in the middle cerebral artery territory. Circumscribed hypointensity is visualized within the left-sided basal ganglia, consistent with a hematoma. (b) The infarct core can be clearly seen on diffusion-weighted imaging (DWI) with similar bleeding-related, central hypointensity within the left-sided basal ganglia. (c) The hemorrhage is best seen on susceptibility-weighted imaging (SWI). (d) Around the hematoma, there is a marked increase in cerebral blood flow (CBF), as seen on the CBF map derived from pseudo-continuous arterial spin labeling (pCASL). Of note, within the hematoma, pCASL-derived CBF maps indicated decreased CBF. (e) For the analyses of the study, any bleeding was excluded from the infarct masks during image segmentations.

**Figure 3.**
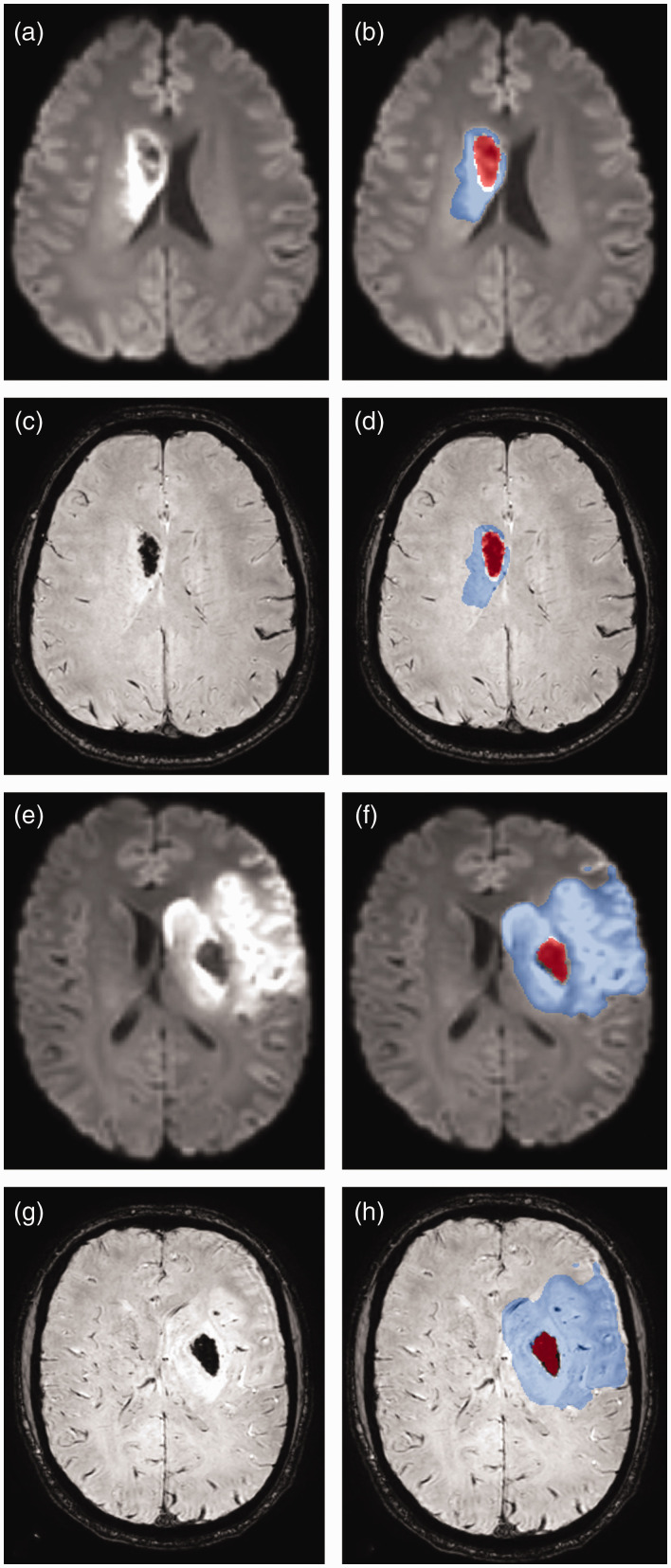
Two exemplary cases of parenchymal hematoma (PH) after thrombectomy. The left column shows co-registered diffusion-weighted imaging (DWI, a, e) and susceptibility-weighted imaging (SWI, c, g) sequences. The right column shows the manually segmented masks for both the infarct (in blue; infarct segmentations of DWI-positive ischemic lesions) and the hematoma (in red) overlaid onto DWI (b, f) and SWI (d, h) sequences. The second case (bottom two rows, e–h) is the same as in Figure 2.

Perfusion imaging by pCASL is a T2*-based modality, therefore signal changes caused by bleeding are highly likely. To avoid this, any bleeding that was visible on SWI was excluded from the infarct mask. Co-registered SWI data were available to aid in all segmentations. An exemplary case of a patient with HT is illustrated in Figure 2. Hematoma on SWI sequences in patients with HI1, HI2, PH1, or PH2 was segmented by two blinded raters (MRHP, 3 years of experience; JB, 1 year of experience), and examples of infarct and hematoma masks overlaid onto DWI and SWI data can be found in Figure 3.

### No-reflow subcohort

No reflow was defined as visible reduction of CBF in ≥50% volume of the infarct territory as well as a <−15%CBF difference for the entire infarct despite near complete or complete recanalization (eTICI 2c or 3), which is in agreement with the definition in the meta-analysis by Ng et al.^36^ A threshold for visual grading of ≥50% volume was applied in order to enhance the discernment of genuine perfusion changes, given that compared to the publication by Ng et al. reporting on contrast agent-based MRI perfusion or CT perfusion (CTP) imaging,^36^ CBF from pCASL was used in the present study, which has lower SNR and is more susceptible to artifacts. Visual assessment was performed by two independent raters (MRHP, 3 years of experience; JB, 1 year of experience), who were blinded to all other variables. A total of 10 patients with no-reflow phenomenon were identified. This subcohort was compared to the rest of the cohort (Table 1).

### Statistical analysis

Statistical analyses were performed with SPSS (version 29; IBM Corp., Armonk, NY, USA). Continuous and ordinal variables are given as median and interquartile range (IQR). Categorical variables are reported as absolute and relative frequencies.

The cohort was split into two groups based on outcome: patients with functional independence (mRS ≤2 at 3-months FU examinations) and patients without functional independence (mRS >2 at 3-months FU examinations). All cohort variables were compared between these two groups. Similarly, the cohort was split into patients with and without no-reflow phenomenon. All variables were compared across these subcohorts in a similar manner. The Mann-Whitney U test was used to compare continuous and ordinal variables. The Fisher's exact test was used to compare categorical variables, as all were distributed on a 2 × 2 crosstab.

All variables that were found to be significantly associated with functional independence in univariate analysis were entered into a mulitvariate logistic regression model to predict functional independence using a stepwise forward variable selection method. To predict occurrence of the no-reflow phenomenon, all variables that were significantly associated with no reflow in univariate analysis were entered into a multivariate logistic regression model using an enter variable selection method. The odds ratio (OR) and 95% confidence intervals (CIs) were computed for the regression models. P-values below 0.05 were considered statistically significant.

## Results

### Baseline and outcome data

A total of 111 patients treated with MT for ischemic stroke due to LVO of the anterior circulation were included. Post-stroke MRI acquisitions took place in the acute post-stroke phase at median 4 days (IQR 3–5 days) after the intervention. The median age was 74 years (IQR 61–82 years), and 45% of patients (n = 50) were male.

Median pre-stroke disability scores (before symptom onset) as estimated by mRS amounted to 0 (IQR 0–1). The median admission NIHSS score was 12 (IQR 7–16). The most frequent risk factors of stroke were a history of hypertension in 70% of patients and atrial fibrillation in 53% of patients. The most frequent stroke etiology as classified by the TOAST criteria was cardio-embolism in 50% of patients (n = 55). The median time from symptom onset or last seen well to groin puncture was 290 min (IQR 184–562 min). Furthermore, 42% of patients (n = 47) received pre-interventional intravenous thrombolysis, and 78% of patients (n = 86) were recanalized near completely or completely, achieving an eTICI score of 2c or better. Overall, HT occurred in 44% of patients of the cohort (n = 49), and most hemorrhages were categorized as HI1 (25% of patients). Average hematoma volume of PH1/2 bleeds was 32 ml (IQR 6–53 ml). A spatial overlap between the segmentation masks of the hematoma and the segmentation masks of the infarct was not observed in any of the included patients. Table 1 provides an overview of baseline variables.

The cohort was split into patients who were and were not functionally independent at three months (mRS ≤2 vs mRS >2). The results of group comparisons are shown in Table 1. The following variables were significantly associated with functional independence: age (p = 0.04), pre-stroke mRS (p < 0.001), NHISS at admission (p = 0.03), ASPECTS (p = 0.02), recanalization success of eTICI of 2c or 3 (p = 0.03), infarct volume (p < 0.001), and %CBF difference (p = 0.04). These variables were used in a multivariate regression model to predict functional independence.

In-hospital death occurred in 5 patients (5%). Functional independence (mRS of 2 or below at 3-months FU examinations) was found in 59% of patients. These outcome variables are shown in Table 1.

### Associations of CBF changes with clinical outcome, angiographic outcome, and HT

**Figure 4.**
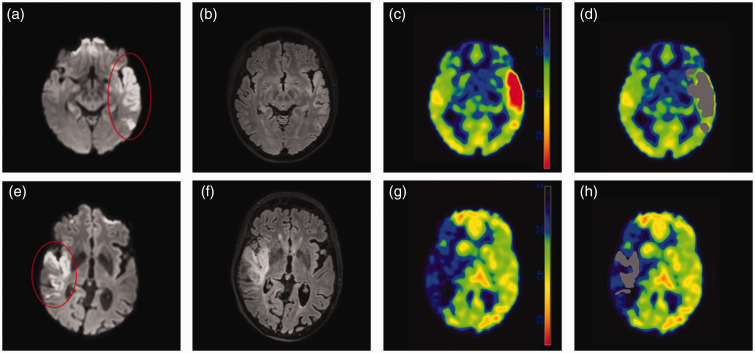
Exemplary patient data. Diffusion-weighted images (DWI, a, e), fluid-attenuated inversion recovery images (FLAIR, b, f), and cerebral blood flow (CBF, c, d, g, h) maps are shown for two exemplary patients. Additionally, DWI-derived masks (in grey) are overlayed onto CBF maps (d, h), indicating the volume of interest in which the CBF map was evaluated for further analyses. The color-coded bands for units in c and g are ml_Blood_/100 g_Tissue_/min. *Patient 1 (a–d):* A 61-year-old woman with a history of sudden onset facial paresis and dysarthria (baseline National Institutes of Health Stroke Scale [NIHSS]: 6). Thrombectomy was performed successfully with an expanded Treatment in Cerebral Infarction (eTICI) 3 result. (a) DWI demonstrates the infarct focus at the left insula and temporal lobe (red circle). (b) On the FLAIR image, subtle parenchymal hyperintensity (swelling) at the left temporal cortex is seen. (c) Five days after thrombectomy, perfusion increase appears at the corresponding left middle cerebral artery (MCA) territory using pseudo-continuous arterial spin labeling (pCASL). *Patient 2 (e–h):* A 77-year-old woman with a history of sudden onset left brachiocephalic hemiparesis, dysarthria, and left-sided neglect (baseline NIHSS: 7). Thrombectomy was performed successfully with an eTICI 3 result. (e) DWI demonstrates the infarct focus at the right insula and temporal lobe (red circle). (f) On the FLAIR image, parenchymal hyperintensity is shown in the corresponding area. (g) Five days after thrombectomy, pCASL shows hypoperfusion at the right MCA territory. This is an exemplary case of the no-reflow phenomenon.

Post-stroke MRI revealed a median infarct volume of 51 ml (IQR 13–136 ml). Infarct core perfusion as measured by pCASL-based CBF showed a median %CBF difference compared to the contralateral unaffected side of 16% (IQR −9–51%). Figure 4 shows imaging data from two exemplary patients, on the upper row a patient with perfusion increase and on the lower row a patient with hypoperfusion following MT; the rightmost images show an overlay of the infarct mask with the CBF maps from pCASL.

**Figure 5.**
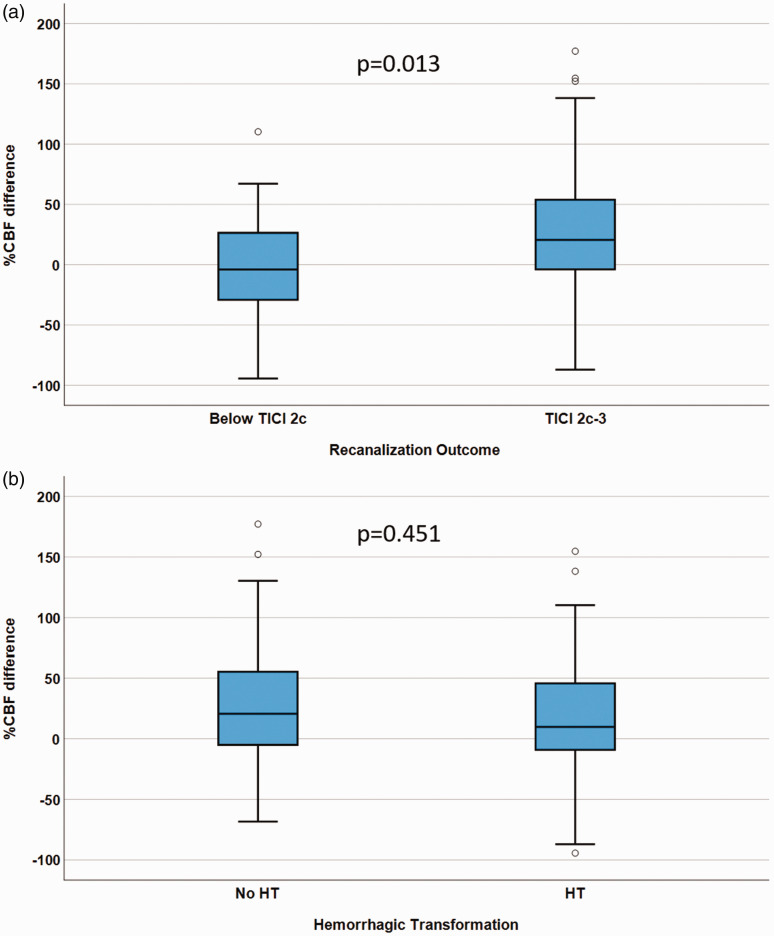
Relationship between infarct percent cerebral blood flow (CBF) difference/angiographic outcome, and %CBF difference/hemorrhagic transformation (HT). Box plots with black bars indicating medians, showing the association between %CBF difference in patients with expanded Treatment in Cerebral Infarction (eTICI) 2c-3 vs. eTICI ≤2 b (a), and in patients with HT vs. patients without HT (b).

Post-stroke infarct CBF was significantly higher in patients with near complete or complete recanalization than in patients without near complete or complete recanalization (median %CBF difference eTICI2c/3 = 20.6% [IQR −4.2–54.3%] and ≤eTICI2b = −3.9% [IQR −32.7–28.2%], p = 0.013; Figure 5(a)). The %CBF difference was similar between patients with and without HT (median %CBF difference in patients without bleeding 20.6% [IQR −6.4–56.19%] and 9.8% [IQR −9.4–46.2%] in patients with HT, p = 0.451; Figure 5(b)). There was no significant difference in %CBF difference between patients with PH1/PH2 HT and all other patients (median %CBF difference in patients without PH1/2 = 15.8% [IQR −9.1–50.6%] and 33.6% [IQR −9.1–110.3%] in patients with PH1/2, p = 0.29; Supplementary Figure S1a; n = 11 in the PH group). Similarly, there was no significant difference in %CBF difference between patients with SH and all other patients (median %CBF difference in patients without SH = 17.7% [IQR −6.8–52.0%] and −9.7% [IQR −27.9–33.6%] in patients with SH, p = 0.35; Supplementary Figure S1b; n = 3 in the SH group).

### Multivariate regression model to predict functional independence

The following dependent variables were entered into a multivariate logistic regression model using a stepwise forward variable selection method to predict functional independence at 3-months FU examinations: age, pre-stroke mRS, NHISS at admission, ASPECTS, recanalization success of eTICI of 2c or 3, infarct volume, and %CBF difference.

In multivariate regression, %CBF difference (OR = 1.01, 95%CI = 1.002–1.025, p = 0.02), pre-stroke mRS (OR = 0.29, 95%CI = 0.15–0.55, p < 0.001), and infarct volume (OR = 0.99, 95%CI = 0.986–0.996, p < 0.001) were significantly associated with functional independence at 3-months FU examinations. The variables age, NIHSS at admission, ASPECTS, and eTICI 2c/3 were not independently associated with functional independence at three months (p > 0.05). Table 2 shows a summary of multivariate regression analysis results.

**Table 2.** Multivariate logistic regression analysis for functional independence shows factors associated with modified Rankin Scale (mRS) scores of 0–2 at 3-months follow-up (FU) examinations.

Multivariate Analysis	OR	95% CI	p
% CBF difference	1.013	1.002–1.025	**0.024**
Pre-stroke mRS	0.286	0.148–0.550	**<0.001**
Infarct volume	0.991	0.986–0.996	**<0.001**
Age	*	*	0.156
NIHSS at admission	*	*	0.083
ASPECTS	*	*	0.518
eTICI 2c/3	*	*	0.100

OR: odds ratio; CI: confidence interval; CBF: cerebral blood flow; mRS: modified Rankin Scale; NIHSS: National Institutes of Health Stroke Scale; ASPECTS: Alberta Stroke Program Early CT Score; eTICI: expanded Treatment in Cerebral Infarction.

* : no odds ratio (OR) is available for variables removed by the stepwise forward variable selection method.

### No-reflow phenomenon

A subcohort 10 patients with no-reflow phenomenon (visible hypoperfusion in >50% volume of the infarct territory and %CBF difference <−15 despite eTICI 2c or 3 angiographic outcome) was identified (Table 1). Patients with no reflow were significantly older than patients without no reflow (median age no-reflow = 85 years [IQR 71–91 years] and 72 years [IQR 61–81 years] in the rest of the cohort, p = 0.016). Patients with no reflow were significantly more likely to be pre-medicated with platelet aggregation inhibitors (n = 6 [60%] in the no-reflow group and n = 24 [24%] in the rest of the cohort, p = 0.018). The previous two variables were put into a multivariate regression model to predict occurrence of the no-reflow phenomenon.

There was significantly lower %CBF difference in the no-reflow subcohort (median %CBF difference in the no-reflow subcohort = −37% [IQR −51–−18%] and in the rest of the cohort = 20% [IQR −4–53%], p < 0.001). Outcome was significantly worse in the no-reflow subcohort (median mRS at 3 months in the no-reflow subcohort = 5 [IQR 3–6] and median in the remaining cohort = 2 [IQR 1–4], p = 0.002). Similarly, the likelihood of functional independence was significantly lower in patients with no-reflow phenomenon (n = 2 [20%] in the no-reflow subcohort and n = 63 [63%] in the rest of the cohort, p = 0.015). There was a non-significant trend toward more in-hospital death in the no-reflow cohort (n = 2 [20%] in the no-reflow subcohort and n = 3 [3%] in the remaining cohort, p = 0.063). Rates of bleeding as well as SH were similar in both groups.

### Multivariate regression model to predict the no-reflow phenomenon

Age and previous medication with platelet aggregation inhibitors were the only baseline variables significantly associated with the occurrence of the no-reflow phenomenon. Both were included in a multivariate logistic regression model to predict the occurrence of the no-reflow phenomenon (Supplementary Table S1). Only previous medication with platelet aggregation inhibitors was independently associated with the occurrence of the no-reflow phenomenon (OR = 5.4; 95%CI = 1.2–24.1, p = 0.029). Age curtly failed to reach statistical significance as a predictor of the no-reflow phenomenon (OR = 1.07; 95%CI = 0.999–1.15, p = 0.052).

## Discussion

This study examined post-stroke pCASL imaging to assess CBF as an imaging biomarker for functional outcome and HT risk in patients who underwent MT for anterior circulation ischemic stroke. The findings demonstrated that i) increased infarct CBF compared to the unaffected side, along with pre-stroke mRS and infarct volume, predicted functional independence at 90 days post-stroke, ii) there was no significant association between increased infarct CBF and risk of HT, and iii) clinical outcome was diminished in patients with no-reflow phenomenon.

Our study supports Lu et al.'s previous findings, indicating that perfusion increase measured using PASL in the infarct core was associated with higher rates of functional independence 90 days after stroke onset.^13^ Luijten et al. conducted a study using pCASL imaging and found a correlation between relative CBF values and NIHSS scores at 24 hours after onset in 44 patients (40 treated with MT).^12^ Higher relative CBF values in the infarct core were associated with lower NIHSS scores at 24 hours.^12^ However, their analysis was univariate, lacking predictive multivariate regression models and long-term outcome measures.^12^ Yoo et al. studied 51 patients with ischemic stroke treated with MT, using both PASL and pCASL.^37^ They found that increased CBF in the infarct after MT correlated with lower NIHSS scores up to 7 days after stroke, and this change in CBF was a significant predictor of NIHSS scores in a multivariate model.^37^ The studies by Luijten et al. and Yoo et al. were the first to correlate CBF measurements using pCASL with clinical outcomes, focusing on short-term outcomes.^12,37^ In our study, pCASL-derived % CBF increases within the infarct core predicted functional independence at three months after stroke, accounting for potential confounders through multivariate regression analyses. Other predictors of functional independence were infarct volumes and pre-stroke mRS scores, which align with previous studies.^38–40^

The cause of infarct core perfusion increase is a matter of debate, and it may be due to vaso-dilatatory processes due to increased metabolism of the hypoxic tissue or a decrease of vessel wall tonus due to loss of cerebral auto-regulation.^41^ Regardless, it is likely the physiological response to cerebral ischemia-reperfusion injury.^12,13,20,37^ Contrarily, hypoperfusion can result from persistent macrovascular occlusion and microvascular impairments.^13,42,43^ This study found lower relative infarct CBF values associated with angiographic outcomes below eTICI 2c, suggesting inadequate reperfusion due to ongoing (partial) occlusion.

Microvascular impairment, known as the no-reflow phenomenon, can cause decreased perfusion in the infarct area.^44^ This phenomenon may result from tissue edema obstructing capillary beds, pericyte contraction causing microvascular obstruction, or microthrombotic debris from thrombus fragmentation after MT.^25,45,46^ Composition of microthrombi by way of altered local inflammation may play a role in the development of the no-reflow phenomenon.^47,48^ Recent modifications to thrombectomy techniques, such as the use of balloon guide catheters, aim to reduce antegrade blood flow and minimize the risk of microthrombi embolization.^49,50^ Balloon guide catheters have improved patient outcomes and are associated with reduced microstructural impairment in the penumbra.^49–51^ In the Chemical Optimization of Cerebral Embolectomy (CHOICE) trial, intra-arterial thrombolysis after successful recanalization improved patient outcomes by reducing the microthrombus burden in the infarct and penumbra microvasculature.^52^ These clinical findings point to a high relevance of the no-reflow phenomenon after ischemia-reperfusion injury in the brain.^52^

In this study, we identified a small subcohort of 10 patients with the no-reflow phenomenon. These were patients with visible hypoperfusion in >50% volume of the infarct territory despite eTICI 2c or 3 recanalization outcome. This subcohort had significantly worse clinical outcome than the rest of the cohort, which also included cases with suboptimal recanalization outcomes. Both age and previous medication with platelet aggregation inhibitors were significantly associated with the occurrence of the no-reflow phenomenon. A multivariate regression analysis revealed that only use of previous medication with platelet aggregation inhibitors was independently associated with the occurrence of the no-reflow phenomenon, while age barely failed to reach statistical significance within the multivariate model. As platelet aggregation inhibitors are usually prescribed due to atherosclerotic disease, the association between platelet aggregation inhibitors and the no-reflow phenomenon may be explained by a higher (known) atherosclerotic burden in patients with previous use of platelet aggregation inhibitors.^53^ Age, as a general morbidity-accelerating factor, likely also plays a role in the occurrence of the no-reflow phenomenon. Due to the relatively low number of patients with no reflow, partially due to the strict selection criteria for the no-reflow phenomenon in this study, the predictive factors for no reflow need to be further examined in future studies.

Regarding the results related to the no-reflow phenomenon, our findings seem to correspond to the results of the previous meta-analysis of Ng et al.^36^ However, instead of using contrast agent-based MRI perfusion or CTP imaging, we used CBF from pCASL imaging in our study. Specifically, Ng et al. found the no-reflow phenomenon in 33 of 130 patients in a pooled analysis of the Extending the Time for Thrombolysis in Emergency Neurological Deficits—Intra-Arterial (EXTEND-IA), the Tenecteplase Versus Alteplase Before Endovascular Therapy for Ischemic Stroke (EXTEND-IA TNK), and the Determining the Optimal Dose of Tenecteplase Before Endovascular Therapy for Ischemic Stroke (EXTEND-IA TNK Part 2) trials.^4,36,54,55^ Within these trials, patients received either CT-based or DSC-MRI-based perfusion imaging around 24 hours after thrombectomy.^4,54,55^ All patients included in the meta-analysis by Ng et al. had eTICI 2c or 3 angiographic outcomes.^36^ No reflow was defined as rater-identified hypoperfusion in cerebral blood volume (CBV) or CBF within the infarct core and >15% asymmetry compared to the contralateral side.^36^ In our study, the raters were instructed to look for hypoperfusion of ≥50% volume of the infarct territory, a precautionary measure aimed at preventing the inadvertent positive rating of data with lower SNR and potentially higher rates of artifacts as pCASL data (and not contrast agent-based techniques such as DSC-MRI or CTP) were used in the present study. However, following a methodology akin to the meta-analysis conducted by Ng et al., the cases identified by raters exhibited >15% CBF asymmetry when the affected side was compared to the contralateral side (i.e., <−15% CBF difference).^36^ As in our study, the authors found significantly worse clinical outcome in patients with no-reflow phenomenon.^36^ Unlike in our study, the no-reflow cohort was only compared to other patients with eTICI 2c or 3.^36^ The effect seen in our study was strong enough to overcome somewhat worse outcomes produced by suboptimal angiographic results in the comparison group. Although Ng et al. demonstrated nicely the feasibility of screening for the no-reflow phenomenon using DSC-MRI or CTP, in most centers perfusion imaging is not part of the standard post-stroke imaging protocol.^36^ One possible reason for this is the need for administration of contrast media that would otherwise not be required. Our work demonstrates the feasibility of identifying patients with no reflow using pCASL as a contrast media-free method in a real-world study.

In a prior investigation by ter Schiphorst et al. that aimed at identifying the no-reflow phenomenon using ASL, the authors reported observing no reflow in only 1 out of 33 patients.^56^ This particular patient exhibited an excellent outcome, leading the authors to infer that no reflow might be infrequent and might not exert a substantial impact on patient outcomes.^56^ Two key distinctions between our study and this previous study likely account for the disparity in outcomes: firstly, our study encompassed a larger cohort of 111 subjects, thereby providing increased statistical power; secondly, the no-reflow definition criteria in the work by ter Schiphorst et al. were stringent, among others requiring ≥40% hypoperfusion compared to the contralateral side, resulting in only one patient meeting the inclusion criteria (3% vs 9% in our study).^56^

Yu et al. observed a correlation between perfusion increase measured by pCASL and HT in 221 patients with middle cerebral artery (MCA) ischemic stroke, with less than half undergoing MT.^22^ Similarly, Okazaki et al. reported an association between post-stroke perfusion increase measured by PASL and 123I-iodoamphetamine single-photon emission CT and HT in a smaller study of 31 patients.^21^ In our study, we did not find a significant difference in relative infarct CBF values between patients with and without HT. Median relative CBF values were even lower in patients with HT, although not statistically significant. Our study exclusively focused on MT patients, while the work by Yu et al. and Okasaki et al. included conservatively managed MCA stroke patients.^21,22^ In the work by Yu et al., MT was more frequently performed in patients who developed HT, and patients with HT had larger infarct volumes.^22^ Our study was unable to confirm a relationship between pCASL-derived CBF increase in the infarct territory and risk of bleeding.

A potential key advantage of pCASL is its reliance on magnetically labeled arterial blood as an endogenous tracer, eliminating the requirement for exogenous contrast agents and the associated risks.^57,58^ Presently, perfusion-weighted imaging for prognosis estimation is not a standard sequence in post-stroke imaging protocols. We posit that, with anticipated technological advancements addressing artifact susceptibility and required scan durations, potentially along with the wide implementation of a multi-delay ASL sequence, ASL-based perfusion imaging holds promise as the future standard for routine screening for the no-reflow phenomenon in clinical practice and research. Hence, perfusion imaging using pCASL has become a valuable addition to the post-MT MRI protocol at our center. Color-coded CBF maps can be made readily available by the time the patient exits the MRI scanner and can be used quickly to assess the cerebral perfusion status, especially the infarct perfusion. It provides additional information that other, standard post-stroke MRI sequences do not yield.

### Limitations

First, this study is limited by its single-center design. Second, only patients who received a post-stroke MRI examination were included in this study. A potential selection bias towards patients with better outcome cannot be excluded as these patients may be somewhat more likely to be able to undergo post-stroke imaging, while others may have been excluded due to motion artifacts. Third, the herein applied pCASL protocol used a single PLD and is therefore not sensitive to variations in the arrival time of the labeled blood (e.g., due to variance in cardiac output or degree of perfusion from late-arriving collaterals). Residual intravascular tag in the sulcal spaces was addressed by reading the ASL signal within a GM probability mask at a 0.6 threshold, thus limiting high signal spill-over from vessels in the subarachnoid space and low signal from the white matter. However, as GM probability masking is highly dependent on co-registration quality and generally an imperfect practice, incorrect signal spill-over into the region of interest (i.e., cortical band of the infarct core) cannot be excluded. Using a pCASL pulse sequence that incorporates several PLDs might enhance the precision of CBF assessment in future studies. Furthermore, ASL readouts are subject to high variability, making relative ASL measurements essential. This particular trait of ASL-based perfusion imaging may limit the clinical applicability on an individual patient basis, and confirmatory studies of these findings from other centers and scanners are required. Fourth, as a quality control step, GM masks were implemented before extracting CBF data. However, it is likely that this step does not entirely account for substantial alterations in brain symmetry caused by pronounced post-infarct edema and swelling, thereby limiting the accuracy of findings in affected patients. Future investigations should explore and refine data processing methodologies to effectively manage the impact of relevant tissue swelling.

## Conclusion

The %CBF difference, as measured by pCASL at median four days after LVO of the anterior circulation with subsequent MT, may be a viable image-based biomarker for post-stroke clinical outcome. Infarct perfusion increase (infarct CBF greater than in contralateral unaffected hemisphere, %CBF difference >0) is a physiological process that occurs after ischemic stroke, and is likely a surrogate of healthy infarct micro-circulation as well as of successful recanalization. Infarct %CBF difference was associated with favorable clinical outcome. Infarct perfusion increase was not associated with an increased risk of HT in this study. Furthermore, pCASL-based CBF assessment may be suitable to screen for the presence of the no-reflow phenomenon and thus may be used to detect micro-circulatory impairment in the future, pending confirmatory studies.

## Supplemental Material

sj-pdf-1-jcb-10.1177_0271678X241267066 - Supplemental material for Cerebral blood flow from arterial spin labeling as an imaging biomarker of outcome after endovascular therapy for ischemic stroke Supplemental material, sj-pdf-1-jcb-10.1177_0271678X241267066 for Cerebral blood flow from arterial spin labeling as an imaging biomarker of outcome after endovascular therapy for ischemic stroke by Moritz R Hernandez Petzsche, Johannes Bürkle, Gabriel Hoffmann, Claus Zimmer, Sebastian Rühling, Julian Schwarting, Silke Wunderlich, Christian Maegerlein, Tobias Boeckh-Behrens, Stefan Kaczmarz, Maria Berndt-Mück and Nico Sollmann in Journal of Cerebral Blood Flow & Metabolism
