# Supplementary material for: Cerebral blood flow from arterial spin labeling as an imaging biomarker of outcome after endovascular therapy for ischemic stroke
Source: J Cereb Blood Flow Metab. 2024 Oct 4;45(2):219–32. doi: 10.1177/0271678X241267066 (PMC11563528; doi:10.1177/0271678X241267066)
Supplement: sj-pdf-1-jcb-10.1177_0271678X241267066 - Supplemental material for Cerebral blood flow from arterial spin labeling as an imaging biomarker of outcome after endovascular therapy for ischemic stroke [file sj-pdf-1-jcb-10.1177_0271678X241267066.pdf]

Cerebral blood flow from arterial spin labeling as an imaging biomarker of outcome after endovascular therapy for ischemic stroke

Supplementary Material

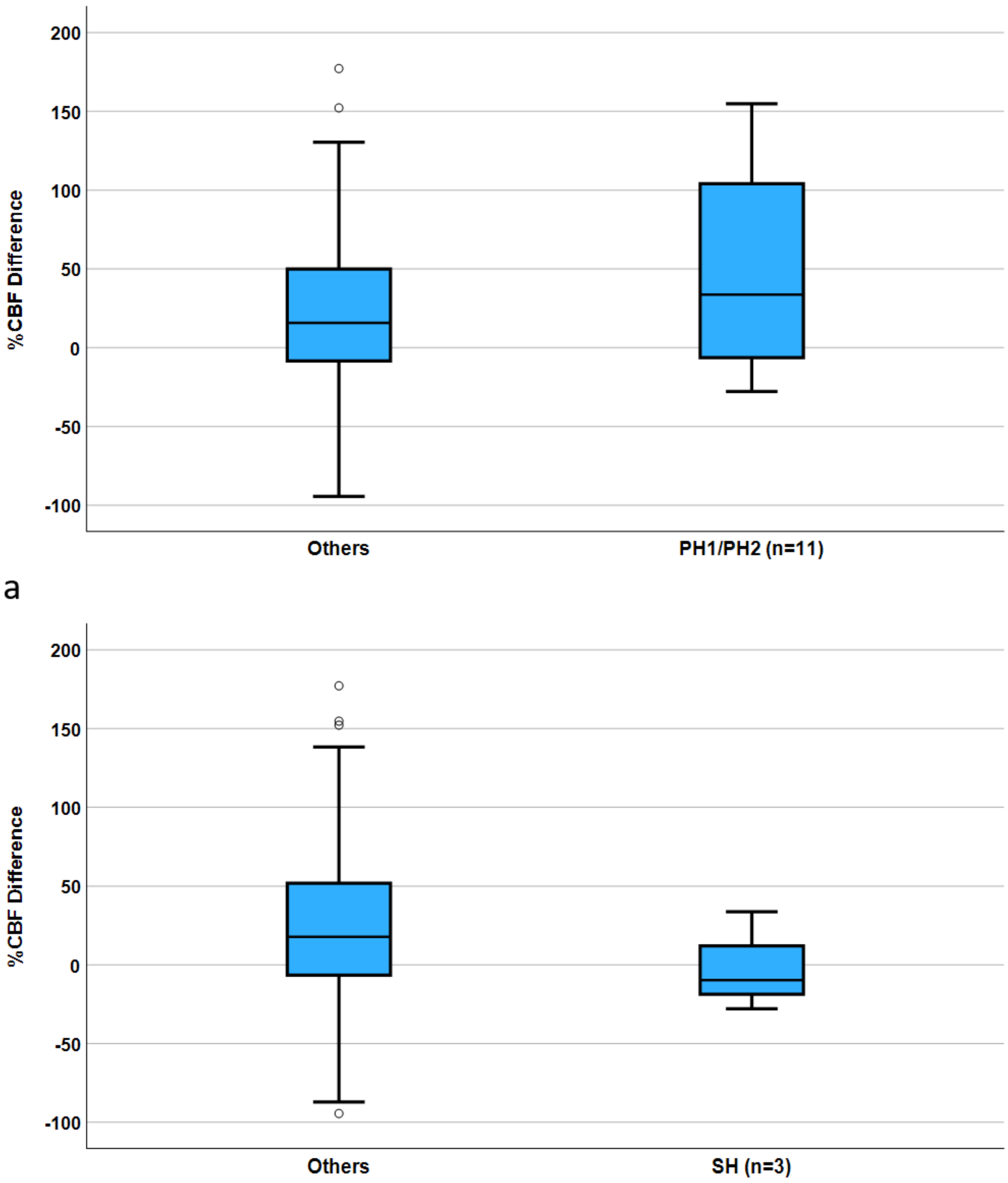

a

b

Supplementary Figure S1. a Comparison of % cerebral blood flow (CBF%) difference of patients with parenchymal hematoma (PH; PH1 or PH2 according to the European Cooperative Acute Stroke Study [ECASS II] classification) and the rest of the cohort. No statistically significant difference was found between the groups (p=0.29). **b** Comparison of CBF% difference of patients with symptomatic hemorrhage (SH) and the rest of the cohort. No statistically significant difference was found between the groups (p=0.35).

Multivariate Analysis	OR	95% CI	p=
**Age**	1.073	0.999-1.152	0.052
**Platelet aggregation inhibitors**	5.353	1.188-24.115	**0.029**

Supplementary Table S1. Multivariate logistic regression analysis to predict the occurrence of no-reflow phenomenon. Both age and platelet aggregation inhibitors, the only variables to be associated with the presence of the no-reflow phenomenon in univariate analysis, were included in a multivariate logistic regression model to predict the occurrence of the no-reflow phenomenon. OR = odds ratio, CI = confidence interval.
